# Chloroform in a Case of Poisoning by Strychnine

**Published:** 1851-08

**Authors:** 


### PATHOLOGY AND PRACTICE OF MEDICINE.

*Chloroform in a Case of Poisoning by Strychnine.*—Mr. G——, aged about 40, of intemperate habits, took from among some medicines, on the 5th inst., a bottle of strychnine; and supposing it to be morphine, as he said, swallowed a dose supposed to be about one or two grains. In about twenty minutes afterwards Dr. Munson was requested to see him, as he was supposed to be in a "fit." He found him in the following condition:—The whole muscular system rigid; the muscles of the back, and of the upper and lower extremities, rigidly contracted; the head drawn back; articulation difficult; sense of tightness about the chest, perspiration flowing profusely from the face and chest. A number of the physicians of the place came to his assistance. The usual remedies recommended in such cases were resorted to, but without any mitigation of the urgent symptoms. The patient was failing rapidly under the increasing spasmodic action of the whole muscular system. It was now determined to administer chloroform, as death was apparently certain without some relief. One drachm of chloroform was put upon a silk handkerchief, and the patient directed to inhale it. The effect was decisive. The patient (who was at this time in a sitting posture, held so by assistants, who could not move him in the least degree without exciting the most frightful and alarming spasms) requested to be placed in a recumbent position, which was done without exciting the least spasm. The chloroform was carefully administered for some hours, the patient holding the handkerchief most of the time himself, in order, as he said, "to keep off the dreadful spasms." From this time he recovered rapidly, and on the 7th instant was able to leave for home, a distance of six or seven miles.—*Boston Med. Jour.*

